# Delayed and immediate cutaneous adverse events during pembrolizumab combination chemotherapy against cervical cancer: Case series

**DOI:** 10.1111/1346-8138.17521

**Published:** 2024-11-11

**Authors:** Takeya Adachi, Tomoya Matsui, Utako Okata‐Karigane, Chiaki Takahashi, Umi Tahara, Mari Hyodo, Akihiro Miyagawa, Kenta Kobayashi, Yoshio Nakamura, Takeru Funakoshi, Hiroshi Nishio, Wataru Yamagami, Hayato Takahashi

**Affiliations:** ^1^ Department of Dermatology Keio University School of Medicine Tokyo Japan; ^2^ Allergy Center Keio University Hospital Tokyo Japan; ^3^ Department of Medical Innovation and Translational Medical Science, Graduate School of Medical Science Kyoto Prefectural University of Medicine Kyoto Japan; ^4^ Department of Obstetrics and Gynecology Keio University School of Medicine Tokyo Japan

**Keywords:** anaphylaxis, cervical cancer, erythema multiforme, immune checkpoint inhibitor, pembrolizumab

## Abstract

Immune checkpoint inhibitors (ICIs), such as pembrolizumab (PEM), are widely recognized for their antitumor efficacy, but they can also lead to various cutaneous adverse events (CAEs). While most CAEs can be managed with topical corticosteroids, severe cases may necessitate halting immunotherapy. The incidence of severe CAEs is notably higher in combination therapies involving ICIs than in monotherapies, emphasizing the need for stringent, long‐term management strategies. This includes potential modifications or discontinuations of the combination therapy. PEM, when added to the conventional paclitaxel + cisplatin (or carboplatin) ± bevacizumab regimen, has shown significant improvements in overall and progression‐free survival for patients with Stage IVB metastatic or locally uncontrolled recurrent cervical cancer. This case series retrospectively examined the incidence and management of CAEs in 19 female patients treated with this combination therapy between October 2022 and May 2023. Four patients exhibiting CTCAE grade 3 were identified. The four cases of severe CAEs involved erythema multiforme after the initial course of PEM combination chemotherapy. Notably, three patients experienced immediate hypersensitivity reactions, including anaphylaxis, during subsequent treatments. This observation underscores the necessity for rigorous dermatological monitoring of patients undergoing PEM combination chemotherapy. Such vigilance is crucial for early detection of adverse reactions and timely adjustment of treatment regimens, thereby enhancing patient safety.

## 1 INTRODUCTION

Immune checkpoint inhibitors (ICIs), including pembrolizumab (PEM), have broad antitumor activity but can cause various cutaneous adverse events (CAEs). Although most CAE cases are mild and can be treated with topical corticosteroids (TCS),^1^ severe CAEs may require the interruption of continuous immunotherapy.^2^ Furthermore, combination therapies involving ICIs have been associated with more severe CAEs compared with monotherapies.^3^ This necessitates more rigorous and long‐term management strategies, including modification to or discontinuation of the combination therapy.

**FIGURE 1:**
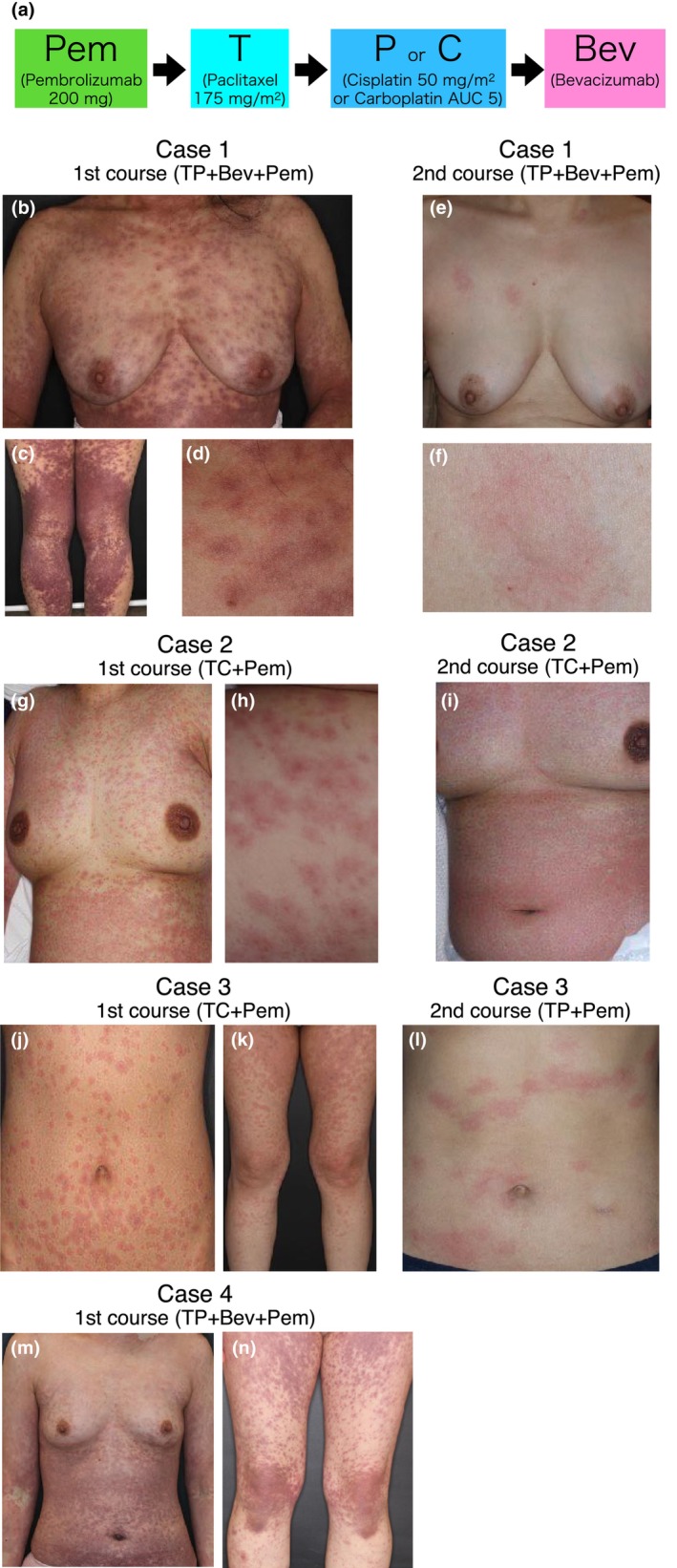
Cutaneous adverse events (CAEs) during pembrolizumab combination chemotherapy against cervical cancer. Regimen overview. (a) Delayed CAEs after the first treatment course. (b–d) Case 1, (g and h) Case 2, (j and k) Case 3, (m and n) Case 4. Immediate CAEs during the second treatment course (e and f) Case 1, (i) Case 2, (l) Case 3.

Recently, the addition of PEM to the conventional paclitaxel + cisplatin (or carboplatin) ± bevacizumab combination therapy was reported to significantly prolong overall and progression‐free survival for Stage IVB metastatic or locally uncontrolled recurrent cervical cancer^4^ (dosage and schedule are described in Figure 1a). To characterize the severe CAEs that can occur during PEM combination chemotherapy, we retrospectively analyzed 19 female patients who received this newly introduced standard regimen from October 2022 to May 2023. The study was approved by the Keio University ethics committee (approval no. 20150197). Using the Common Terminology Criteria for Adverse Events (CTCAE) version 5.0, four patients who exhibited grade 3 symptoms were included in the study.^5^

## 2 CASE REPORTS

Case 1 involved a previously untreated, 55‐year‐old woman with a history of mango allergy who presented with generalized erythema multiforme (EM) and fever 8 days after the first course of paclitaxel + P + BEV + PEM (Table 1, Figure 1b–d). Her symptoms were improved with TCS and oral antihistamines in a week. We selected the same regimen with a reduced dose of paclitaxel and cisplatin for the second course after 28 days, but urticarias appeared during PEM administration. The patient continued treatment after spontaneous resolution; however, urticaria‐like erythema appeared immediately after paclitaxel administration and the treatment was discontinued (Figure 1e,f). PEM was discontinued and the patient continued with paclitaxel + cisplatin + bevacizumab, despite two episodes of mild urticaria‐like erythema. The effect of the treatment on cervical cancer was a partial response (PR).

**TABLE 1:** Characteristics of patients who developed erythema multiforme during pembrolizumab combination chemotherapy against cervical cancer.

	Age	Previous treatment regimen	1st course				2nd course						
			Regimen	Onset of CAE	Symptom (CTCAE grade)	Treatment for CAE	Regimen	Onset of CAE	Symptom (CTCAE grade)	Treatment for CAE	Subsequent regimen	Courses without recurrence	Therapeutic effectiveness
Case 1	55	—	TP + Bev + Pem	Day 8	EM + fever (Grade 3)	TCS + AH	TP + Bev + Pem (reduced TP dose)	During Pem and immediately after T	Urticaria	Discontinued P no Bev AH	TP + Bev (without Pem)	12 additional courses	PR (at the 14th course)
Case 2	47	—	TC + Pem	Day 9	EM + fever (Grade 3)	TCS + AH	TC + Pem (no change)	Immediately after C	Urticaria‐like erythema	Discontinued C PSL	TP + Pem (C → P)	6 additional courses	PR (at the 6th course)
Case 3	39	C + RTx	TC + Pem	Day 10	EM + fever (Grade 3)	AH	TP + Pem (C → P)	Immediately after P	Anaphylaxis (Urticaria + dyspnea + vomiting)	Discontinued P Epinephrine	Pem (without TP)	2 additional courses	PD (at the 4th course)
Case 4	38	—	TP + Bev + Pem	Day 7	EM + fever (Grade 3)	C/O	—				TP + Bev + Pem (no change)	11 additional courses	PR (at the 12th course)

Abbreviations: AH, antihistamines; Bev, Bevacizumab; C, Carboplatin; C/O, course observation; CAE, Cutaneous Adverse Events; CTCAE, Common Terminology Criteria for Adverse Events; EM, erythema multiforme; PD, progressive disease; Pem, pembrolizumab; PR, partial response; PSL, prednisolone; RTx, radiation therapy; TC, Paclitaxel Carboplatin; TP, Paclitaxel Cisplatin; TCS, topical corticosteroids.

Case 2 involved a 47‐year‐old woman with no history of previous treatment or allergy who presented with generalized EM and fever 9 days after the first course of paclitaxel + carboplatin + PEM (Table 1; Figure 1g,h). The same regimen was administered in the second course after 24 days, but treatment was discontinued because of urticaria‐like erythema and fever immediately after carboplatin administration (Figure 1i). In the third course, carboplatin was replaced with P, and paclitaxel + cisplatin + PEM was administered instead. The patient continued the treatment without symptom flare‐ups. The effect of the treatment on cervical cancer was PR.

Case 3 involved a 39‐year‐old woman with no history of allergy who had been previously treated more than six times with carboplatin + radiotherapy. Ten days after the first course of paclitaxel + carboplatin + PEM, the patient presented with generalized EM and fever (Table 1; Figure 1j,k). She was treated with antihistamines, and her symptoms resolved within a week. The patient received paclitaxel + cisplatin + PEM for the second course after 28 days and developed urticaria, respiratory distress, vomiting, and abdominal pain immediately after cisplatin administration, which was then discontinued (Figure 1l). In the third course, PEM was administered alone, and the patient continued the treatment without recurrence. The treatment outcome for cervical cancer was progressive disease.

Case 4 involved a 38‐year‐old woman with no history of prior treatment or allergy who presented with generalized EM and fever 7 days after the first course of paclitaxel + cisplatin + bevacizumab + PEM (Table 1, Figure 1m,n). She did not require any specific treatment, and her symptoms improved in 1 week; therefore, the same regimen was continued for the second course after 22 days and beyond, without any recurrence. The effect of treatment on cervical cancer was PR.

## 3 DISCUSSION

Table 1 summarizes information on these four cases. All the patients were female, aged 38–55 years, and exhibited delayed reactions (EM, CTCAE grade 3) that initially developed 7–10 days after the first treatment course. Previous reports have documented EM caused by paclitaxel or PEM,^6^, ^7^ and both drugs were used in all four cases in this study. Since the symptoms did not appear after re‐administration of the same drug combination, the possibility of non‐allergic immune‐related adverse events due to either drug cannot be excluded. A PubMed search using the keywords “erythema multiforme” and “pembrolizumab” yielded 31 reports, including Stevens–Johnson syndrome (SJS) and toxic epidermal necrolysis (TEN). After excluding duplicates and reviews, we summarized 28 case reports, including our own (Table 2). Notably, 14 of these 28 cases (50%) developed delayed‐type hypersensitivity reactions during the first course of treatment, suggesting that mechanisms other than allergic hypersensitivity may be involved. Furthermore, no significant differences were observed between the patients who developed rashes during the first course of treatment and those who developed rashes after subsequent courses in terms of age, gender, type of drug eruption, chemotherapy agents combined with PEM, or type of malignancy. All patients received pre‐medication therapy, including dexamethasone, H2 blockers, and aprepitant, which turned out to be negative against lymphocyte transformation tests in several cases.

**TABLE 2:** Cases of delayed and sever cutaneous adverse events during chemotherapy including pembrolizumab.

	Author/year	Age/sex	Onset of CAE (course)	Type of CAE	Concomitant medications	Malignant neoplasm
1	Wang et al./2018	70/M	35	EM		Melanoma
2	Hwang et al./2019	76/M	9	SJS		Melanoma
3	Ambur et al./2021	68/M	6	EM	Cisplatin, pemetrexed	NSCLC
4	Kadoi et al./2024	70/F	5	EM		Anal canal cancer
5	Saw et al./2017	50/F	5	SJS		Nasopharyngeal carcinoma
6	Wang et al./2018	80/F	4	EM		SCC
7	Shazib et al./2020	70/M	4	EM		Oral cavity SCC
8	Shih et al./2021	83/M	4	EM		Bladder cancer, Lung adenocarcinoma
9	Hines et al./2021	72/M	3	Bullous EM		Urothelial cancer
10	Zhang et al./2022	32/M	3	SJS		Hepatocellular carcinoma
11	Sekimata et al./2024	49/F	2	EM	Paclitaxel, carboplatin	Uterine cervical adenocarcinoma
12	Saw et al./2017	53/M	2	SJS		Renal cell carcinoma
13	Alexandris et al./2022	60/M	2	TEN		NSCLC
14	Gallo et al./2023	77/M	2	TEN	FOLFOX, trastuzumab	Esophageal adenocarcinoma
15	Adachi et al./2024	55/F	1	EM	Paclitaxel, cisplatin, bevacizumab	Cervical cancer
16		47/F	1	EM	Paclitaxel, carboplatin	Cervical cancer
17		39/F	1	EM	Paclitaxel, carboplatin	Cervical cancer
18		38/F	1	EM	Paclitaxel, cisplatin, bevacizumab	Cervical cancer
19	Haratake et al./2018	69/M	1	SJS		Lung adenocarcinoma
20	Ryu et al./2022	64/M	1	SJS		Urothelial cancer
21	Shi et al./2023	73/F	1	SJS like		Oral cavity SCC
22	Oguri et al./2021	76/M	1	SJS/TEN		Lung adenocarcinoma
23	Robinson et al./2022	55/F	1	SJS/TEN		Cervical SCC
24	Kumar et al./2019	57/F	1	TEN		Lung adenocarcinoma
25	Cai et al./2019	63/M	1	TEN		Lung adenocarcinoma
26	Kian et al./2022	65/M	1	TEN		NSCLC
27	Alexandris et al./2022	70/F	1	TEN		NSCLC
28	Neema et al./2023	55/M	1	TEN		SCC of the penis

Abbreviations: CAE, cutaneous adverse events; EM, erythema multiforme; FOLFOX, folinic acid, fluorouracil and oxaliplatin; NSCLC, non‐small cell lung cancer; SCC, squamous cell carcinoma; SJS, Stevens–Johnson syndrome; TEN, toxic epidermal necrolysis.

Regarding the immediate hypersensitivity reactions, platinum‐based chemotherapeutic agents are well‐documented triggers.^8^ In this study, Cases 2 and 3 exhibited immediate symptoms concurrent with platinum drug administration during the second course, which resolved after drug discontinuation, implicating them as potential causative agents. Previous reports have noted that the risk of allergic reactions increases after more than five administrations of platinum‐based drugs.^9^ In Case 3, where the patient experienced anaphylaxis, there was a history of six prior cisplatin administrations, which may have contributed to the increased risk of a hypersensitivity reaction. In Case 1, urticarial‐like rashes appeared immediately after the administration of paclitaxel, and similar but milder rashes occurred with subsequent treatments, suggesting paclitaxel as the causative agent in this case. There have been no previous reports of immediate‐type reactions secondary to EM during PEM combination chemotherapy, and the exact mechanism remains unknown. However, there is a documented case of a delayed hypersensitivity reaction to an antimicrobial agent followed by an immediate reaction to another antibiotic in a tuberculosis patient on multidrug therapy.^10^ This suggests that similar rare occurrences may occur in patients undergoing repeated courses of multiple treatments.

The incidence and severity of skin rashes have been reported to increase when ICIs, including PEM, are used in combination with conventional cytotoxic anticancer therapies.^8^ In this study, the incidence of CTCAE grade 3 symptoms was 22.2% (4/19), which is significantly higher than the 4.6% reported in the KEYNOTE‐826 study and the 8.6% in the Japanese subset.^3^, ^4^ However, in all four of our cases, chemotherapy was continued with almost the same drug combination without recurrence of EM, suggesting that these reactions may be triggered by non‐allergic mechanisms, and re‐administration can be considered with careful monitoring. A Japanese study that included 11 cases of skin rash following combined ICI therapy showed that the symptoms could be managed with TCS alone, allowing the continuation of the same treatment regimen in nearly all cases, even if the suspected drug was reintroduced after recovery of the skin rash.^11^ While there are no reports of re‐administration following SJS or TEN, and these conditions are more severe than EM, cautious delabeling could be considered in selected cases. Of note, in our study, immediate reactions at subsequent doses necessitated regimen alterations, and systemic corticosteroid administration was required in one instance. This study lacks comparative analysis and more diverse and multicenter patient cohorts could help to validate and expand these findings.

In conclusion, this study reports four cases of CAE associated with pembrolizumab combination chemotherapy in patients with metastatic or recurrent cervical cancer (Figure S1). All the cases developed EM after the initial course, with three experiencing immediate hypersensitivity reactions, including anaphylaxis, which occurred during the subsequent administrations, likely triggered by cytotoxic agents such as cisplatin or paclitaxel. Therefore, patients presenting with EM after pembrolizumab combination chemotherapy could potentially continue the same regimen but must be placed under stringent dermatological surveillance. This approach can identify the potential onset of immediate reactions and ensure prompt treatment regimen adjustments to maintain patient safety.

## FUNDING INFORMATION

This work was partially supported by the Scientific Research Fund of the Ministry of Health, Labour and Welfare, Japan [Grant Number: 21FE2001]; the JSPS KAKENHI [Grant Number: 22K16268]; and the Japan Agency for Medical Research and Development (AMED) [Grant Numbers: 23ek0410090, 23ek0410106].

## CONFLICT OF INTEREST STATEMENT

The authors declared no Conflict of interest.

## DECLARATION OF GENERATIVE AI AND AI‐ASSISTED TECHNOLOGIES IN THE WRITING PROCESS

During the preparation of this work the authors used ChatGPT 4.0/OpenAI, Microsoft Corporation in order to improve language and readability. After using this tool/service, the authors reviewed and edited the content as needed and take full responsibility for the content of the publication.

## Supporting information


**Figure S1.** Strategic approach for managing cutaneous adverse events (CAE) associated with pembrolizumab combination chemotherapy in patients with metastatic or recurrent cervical cancer.
