# Supplementary figures and images for: Delayed and immediate cutaneous adverse events during pembrolizumab combination chemotherapy against cervical cancer: Case series
Source: J Dermatol. 2024 Nov 11;52(1):132–7. doi: 10.1111/1346-8138.17521 (PMC11700920; doi:10.1111/1346-8138.17521)

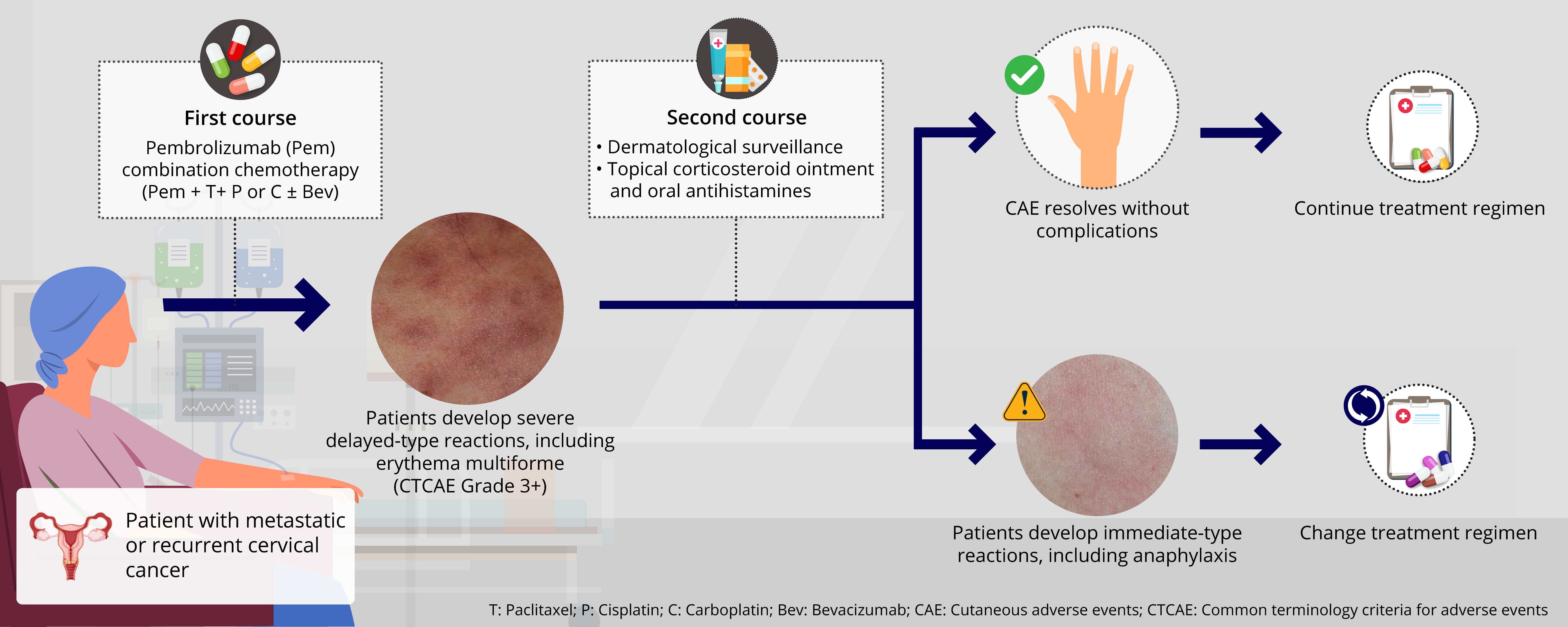

Supplement: Supplementary file 1 — Figure S1. Strategic approach for managing cutaneous adverse events (CAE) associated with pembrolizumab combination chemotherapy in patients with metastatic or recurrent cervical cancer. [file JDE-52-132-s001.tif]
